# Reduced tillage and crop diversification can improve productivity and profitability of rice-based rotations of the Eastern Gangetic Plains

**DOI:** 10.1016/j.fcr.2022.108791

**Published:** 2023-02-01

**Authors:** Muhammad Arshadul Hoque, Mahesh K. Gathala, Jagadish Timsina, Md.A.T.M. Ziauddin, Mosharraf Hossain, Timothy J. Krupnik

**Affiliations:** a Farm Machinery and Post-Harvest Process Engineering Division, Bangladesh Agricultural Research Institute (BARI), Gazipur 1701, Bangladesh; b Department of Farm Power and Machinery, Bangladesh Agricultural University, Mymensingh 2202, Bangladesh; c International Maize and Wheat Improvement Center (CIMMYT), Sustainable Agrifood Systems, House 10/B, Road 53, Gulshan-2, Dhaka 1213, Bangladesh; d Global Evergreening Alliance, Burwood East, Melbourne, VIC 3151, Australia

**Keywords:** ANOVA, analysis of variance, AT, alternate tillage, BARC, Bangladesh Agricultural Research Centre, BARI, Bangladesh Agricultural Research Institute, CSISA, Cereal Systems Initiative for South Asia, CA, conservation agriculture, CT, conventional tillage, DFAT, Department of Foreign Affairs and Trade, EGP, Eastern Gangetic Plains, GM, gross margin, GR, gross return, REY, rice equivalent yield, R-M, rice-maize, R-M-MB, rice-maize-mung bean, R-R, rice-rice, R-W, rice-wheat, R-W-MB, rice-wheat-mung bean, SOC, soil organic carbon, SREY, system rice equivalent yield, SRFSI, Sustainable and Resilient Farming Systems Intensification, ST, strip tillage, TAFSSA, Transforming Agrifood Systems in South Asia, TVC, total variable cost, ZT, zero tillage, Alternate tillage, Calorie and protein yields, Conservation agriculture, Gross margin, Labor use, Relative yield change, Cropping systems diversification

## Abstract

Intensive rice (*Oryza sativa*)-based cropping systems in south Asia provide much of the calorie and protein requirements of low to middle-income rural and urban populations. Intensive tillage practices demand more resources, damage soil quality, and reduce crop yields and profit margins. Crop diversification along with conservation agriculture (CA)-based management practices may reduce external input use, improve resource-use efficiency, and increase the productivity and profitability of intensive cropping systems. A field study was conducted on loamy soil in a sub-tropical climate in northern Bangladesh to evaluate the effects of three tillage options and six rice-based cropping sequences on grain, calorie, and protein yields and gross margins (GM) for different crops and cropping sequences. The three tillage options were: (1) conservation agriculture (CA) with all crops in sequences untilled, (2) alternating tillage (AT) with the monsoon season rice crop tilled but winter season crops untilled, and (3) conventional tillage (CT) with all crops in sequences tilled. The six cropping sequences were: rice-rice (R-R), rice-mung bean (*Vigna radiata*) (R-MB), rice-wheat (*Triticum aestivum*) (R-W), rice-maize (*Zea mays*) (R-M), rice-wheat-mung bean (R-W-MB), and rice-maize-mung bean (R-M-MB). Over three years of experimentation, the average monsoon rice yield was 8% lower for CA than CT, but the average winter crops yield was 13% higher for CA than CT. Systems rice equivalent yield (SREY) and systems calorie and protein yields were about 5%, 3% and 6%, respectively, higher under CA than CT; additionally, AT added approximately 1% more to these benefits. The systems productivity gain under CA and AT resulted in higher GM by 16% while reducing the labor and total production cost under CA than CT. The R-M rotation had higher SREY, calorie, protein yields, and GM by 24%, 26%, 66%, and 148%, respectively, than the predominantly practiced R-R rotation. The R-W-MB rotation had the highest SREY (30%) and second highest (118%) GM. Considering the combined effect of tillage and cropping system, CA with R-M rotation showed superior performance in terms of SREY, protein yield, and GM. The distribution of labor use and GM across rotations was grouped into four categories: R-W in low-low (low labor use and low GM), R-M in low-high (low labor use and high GM), R-W-MB and R-M-MB in high-high (high labor use and high GM) and R-R and R-MB in high-low (high labor use and low GM). In conclusion, CA performed better than CT in different winter crops and cropping systems but not in monsoon rice. Our results demonstrate the multiple benefits of partial and full CA-based tillage practices employed with appropriate crop diversification to achieve sustainable food security with greater calorie and protein intake while maximizing farm profitability of intensive rice-based rotational systems.

## Highlights

• Rice-maize (R-M) outperformed over all other rice-based cropping systems considering yields and profits.
• Conservation agriculture (CA) and alternate tillage (AT) outperformed than conventional tillage (CT).
• R-M and rice-wheat-mungbean systems are more beneficial when layered with CA and AT.
• Rice-rice system is more labor intensive and less profitable than other systems.
• CA is good for winter crops and for cropping systems but not for rice but AT can be viable alternative option.

## 1 Introduction

Double cropping of monsoon rice with winter crops (maize, wheat, *boro* rice, pulses, oilseeds and vegetables) or rice-fallow in sequence is commonly practised in south Asia (Timsina et al., 2010, Krupnik et al., 2015). In many areas, especially in the alluvial Eastern Gangetic Plains (EGP) in Bangladesh, eastern India, and eastern Nepal, the third crop of mung bean or jute, or other short-duration crops may also be grown under the triple cropping systems (Timsina et al., 2013;Islam et al., 2019, Gathala et al., 2020a, Gathala et al., 2020b). Among the various rotations*,* rice-rice (R-R), rice-wheat (R-W), and rice-maize (R-M) together cover approximately 75% of the net cultivable area in the EGP (Gathala et al., 2020b). These crops contribute greatly to household food and nutrition security, and livelihoods for low to middle-income rural and urban populations (Chauhan et al., 2011, Timsina et al., 2018, Islam et al., 2019). Due to the immense importance of these cereals and the unavailability of new land for expanding cultivation in the population-dense EGP, it is increasingly crucial to utilize existing dry season fallow land for multiple cropping. There is plenty of scope to intensify the cropping systems and practise triple cropping systems, especially by including short-duration pulses (mung bean), oilseeds (mustard), and fiber crop (jute) in the EGP (Krupnik et al., 2015, Islam et al., 2019, Rashid et al., 2019, Gathala et al., 2020b). Despite their importance for human nutrition, most studies on cropping systems and tillage techniques in south Asia focus on yield improvements alone without considering protein and calorie yields for food-insecure smallholder farming households and rural communities (DeFries et al., 2015).

In the EGP, mainly rice-based cropping systems are practised with intensive tillage (puddling for rice and repetitive tillage in the ensuing winter crops) and with the burning or complete removal of crop residues from the field. Concerns are increasing that intensive tillage practices combined with injudicious use of natural resources and external inputs can negatively influence soil quality, potentially causing soil acidification and loss of soil organic carbon (SOC) (Jat et al., 2018, Sinha et al., 2019, Jat et al., 2020). Repetitive tillage requires intensive water and energy use (Gathala et al., 2013, Gathala et al., 2016, Gathala et al., 2020a, Islam et al., 2019) and emits large amounts of greenhouse gases (Ladha et al., 2015, Kumar et al., 2018, Gathala et al., 2020b). More resource-efficient practices, including zero, reduced/strip tillage, full or partial retention of crop residue, and diversified crop rotations, are widely advocated as options for decreasing the use of non-renewable resources, reversing soil degradation, and restoring soil fertility, while reducing emissions (FAO, 2011, Pretty et al., 2018, Dixon et al., 2020, Gathala et al., 2020a). Conservation agriculture (CA) can facilitate improved crop establishment and timely sowing, maintain or increase yield, lower water and energy use, lower production cost and increase income, and improve the quality of soil, while improving system resilience (Verhulst et al., 2010, Kumar et al., 2019, Gathala et al., 2013, Gathala et al., 2015, Gathala et al., 2016, Gathala et al., 2020b, Jat et al., 2014). Research in south Asia has shown that CA or strip tillage (ST), with residue retention, typically results in greater yields and profits from non-rice crops compared to rice (Erenstein and Laxmi, 2008; Gathala et al., 2015; Islam et al., 2019; Gathala et al., 2021). Farmers survey data also tend to confirm these findings (Keil et al., 2017; Akter et al., 2021, Timsina et al., 2013). In conventional system of rice cultivation, rice seedlings are transplanted into repetitively tilled, puddled, and flooded fields. However, seedlings can also be transplanted without puddling, which can save water, energy, labor, and overall production cost for rice cultivation (Krupnik et al., 2014, Haque et al., 2016, Hossen et al., 2018, Gathala et al., 2021). In practice, executing all the ‘required’ components of CA in rice-based systems where rice and succeeding crops are grown under contrasting environments is challenging. There is an intermediate situation, however; farmers frequently make use of alternating tillage (AT), where monsoon rice is produced with full tillage and the dry winter season crops are established using zero tillage (ZT) or ST, with or without residue retention (Krupnik et al., 2014, Keil et al., 2017).

The most burdensome tasks of manual transplanting in rice and manual seeding or weeding in crops are generally performed by rural women, the degree and intensity of which depends on the region and community; rice production is particularly arduous, time-consuming and resource-demanding (Chhetri et al., 2020, Gathala et al., 2021). There is a potential to eliminate these unwanted tasks and follow CA-based mechanized crop production practices such as non-puddled rice transplanting and ZT/ST seeding. The intensive use of labor and energy under conventional agronomic practices results in high production costs; combined with low crop yields because of inefficient agronomic management, this results in low gross margins (Gathala et al., 2021). Low gross margins and laborious work experienced in agriculture have forced rural male youth to work away from home to meet family household needs. Such rural youth outmigration further aggravates labor crises at crop peak seasons and the cost of hiring additional labor to support those families left behind on the farm and enable their agricultural production to continue (FAO, 2018, Darbas et al., 2020, Gathala et al., 2021).

A few studies (Krupnik et al., 2014, Islam et al., 2019) have evaluated the impacts of different tillage methods under double cropping systems, comparing the full suite of CA practices to AT, in which rice was puddled and winter crops were subjected to ST/ZT. However, there are also no robust studies comparing CA and AT with CT across various rice-based cropping systems; in particular, little work has been done to assess protein and calorie yields in cropping systems under different tillage practices. Further, the labor use and gross margins of the multiple rice-based rotational systems in the EGP have not been examined. An improved understanding of the effects of tillage practices on crop productivity and profitability can help explain their performance and identify the region's most productive and profitable systems. Thus, the objective of this study was to evaluate CA and AT (i.e., CT in monsoon rice and ST with residue retention in winter and spring crops) against CT, under six double and triple cropping systems in terms of grain yield, grain protein and calorie yields, labor use, and gross margin of different crops and cropping systems. Such a study also provides the potential benefit of layering suitable crop rotations and tillage practices on loamy soil in a sub-tropical environment in northern Bangladesh in the EGP, representing a major rice-growing region.

## 2 Materials and methods

### 2.1 Site/soil description

From 2013–2016, a field study was conducted at the Regional Agricultural Research Station of the Bangladesh Agricultural Research Institute (BARI) in the sub-tropical climate of Jamalpur (24° 56' 30.68''N lat., 89° 55' 40.39''E long., 21.6 m) in northern Bangladesh. Jamalpur soils belong to AEZ 9 (Old Brahmaputra Floodplains), which are characterized as raw alluvial, noncalcareous grey soils, known for poor drainage. AEZ 9 is part of the Brahmaputra-Jamuna Floodplains of northern and eastern slopes of the Himalayas and has a catchment area of 583000 km^2^ that covers about 16,436 km^2^ area in Bangladesh. The soil was analysed at BARI for textural class, physical and chemical parameters, following the analytical standard procedure protocols. It was classified as loam (sand, 47.3%; silt, 30.0%; and clay, 22.7% at 0–15 cm soil layer). Initial chemical analysis from 0 to 15 cm soil depth conducted prior to the start of the experimentation in 2013 indicated that the soil of the experimental site was slightly acidic-to-neutral (pH 6.2). It had low soil organic matter (7.7 g kg^−1^), very low total N (0.42 g kg^−1^), low K and B (0.05 meq 100^−1^ ml and 0.14 μg ml^−1^), and medium S and Zn (10.58 μg ml^−1^ and 0.80 μg ml^−1^), but high P (12.8 μg ml^−1^) and very high Cu, Fe and Mn (0.98, 36.0 and 3.7 μg ml^−1^, respectively).

### 2.2 Weather information

**Fig. 1:**
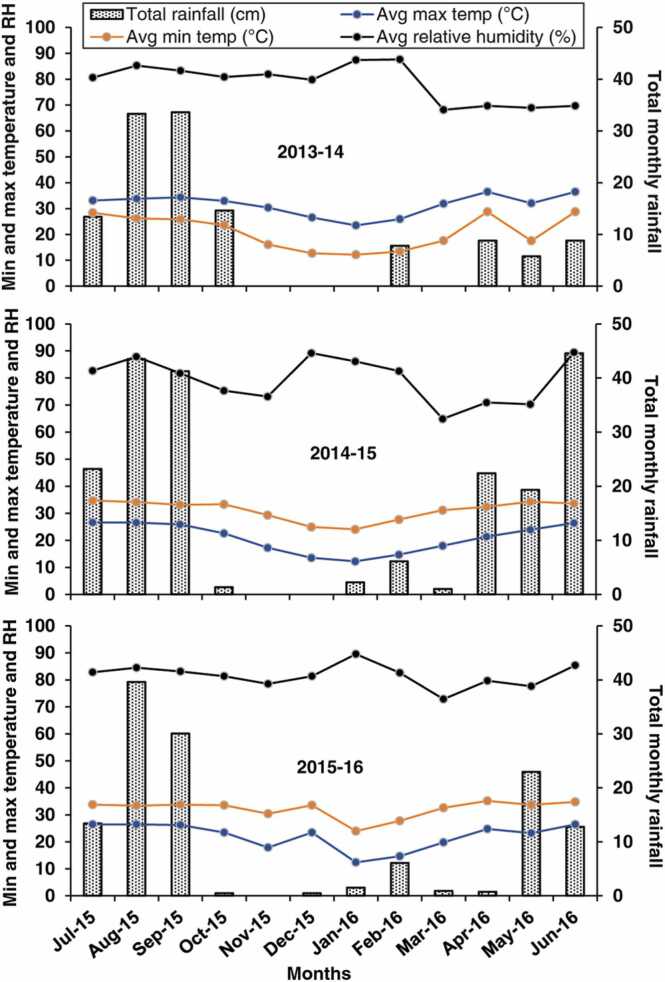
Weather conditions during three years of experimentation in a sub-tropical environment, Jamalpur, Bangladesh (top: 2013–2014; middle: 2014–2015; bottom: 2015–2016).

Weather data for the experimental period were monitored via the weather station installed at the experiment site. Total monthly rainfall in each year varied considerably in amount and distribution. The total rainfall during the monsoon rice season (July–October) varied from 113 cm in 2014 to 83 cm in 2015 (Fig. 1). The maximum and minimum monthly mean temperatures during the rice season ranged from 33.0° to 34.7°C and 22.6–28.4 °C, respectively, while monthly mean relative humidity varied from 75% to 88% across years.

The total rainfall during the winter ‘*rabi*’ season (November to April) ranged from 10 cm in 2013–2014–31 cm in 2014–2015. The maximum monthly mean temperature ranged from 24.1° to 32.4°C (2014–2015) to 23.5–36.4 °C (2013–2014), while the minimum monthly mean temperature ranged from 12.3° to 21.3°C (2014–2015) to 12.2–28.7 °C in 2013–2014. Temperatures were higher in April and May compared to other months. The second year of experimentation (2014–2015) had higher minimum and maximum monthly mean temperatures than the other two years, with the potential for heat stress and grain sterility. The mean relative humidity remained below 70% in the drier months (March to May).

### 2.3 Experimental and treatment details

**Fig. 2:**
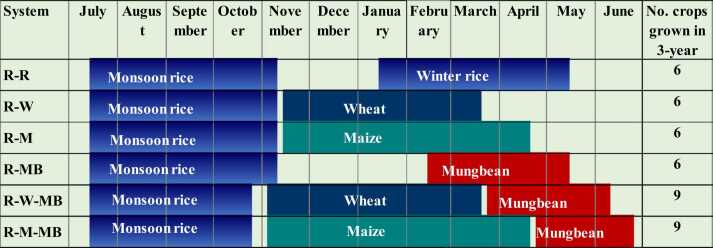
Annual crop calendar for different cropping systems. R-R, R-W, and R-M indicate rice-rice, rice-wheat and rice-maize rotations, while R-MB, R-W-MB and R-M-BM indicate rice-mung bean, rice-wheat-mung bean, and rice-maize-mung bean rotations.

The experiment was conducted in a split-plot design with four replications, where six cropping systems were assigned to the main plots and with three tillage options in the subplots. The main plot size was 20 m × 20 m (400 m^2^) and the subplot size was 20 m × 6 m (120 m^2^). The field experiment was conducted for three years, from monsoon (the ‘*aman’* season) rice in 2013 to mung bean in the spring of 2016. In each annual crop cycle, two to three crops were grown in a sequence in designated cropping rotations (each crop cycle was completed from July to June within a one-year annual crop calendar (Fig. 2)). In three years of experimentation, six and nine crops were grown under double cropping and triple cropping sequences, respectively.

#### 2.3.1 Cropping systems

All six cropping systems treatments had monsoon season rice in common and differed in the winter (*rabi*) crops; two systems (rice-wheat and rice-maize) were further intensified by including a short duration mung bean in the spring season (March-June) as the third crop in one-year crop cycle rotation (Fig. 2). The six cropping systems were monsoon *aman* rice–winter (*boro*) rice (R-R); *aman* rice-wheat (R-W); *aman* rice–maize (R-M); *aman* rice–mung bean (R-MB); *aman* rice–wheat–mung bean (R-W-MB); and *aman* rice–maize–mung bean (R-M-MB).

#### 2.3.2 Three tillage options

The three tillage treatments were conservation agriculture (CA), in which monsoon *aman* rice was transplanted without any preparatory tillage and puddling (wet tillage), while all other crops in rotation were sown under strip tillage (ST) without any prior tillage; alternate tillage (AT) in which monsoon rice was transplanted in puddled soil (CT) while the succeeding winter or spring crops were sown under ST (as with CA); and CT, in which rice (both aman and boro) was transplanted in puddle soil and other winter crops were planted under intensive wet or dry tillage.

In CT, tillage for land preparation for all crops and puddling for rice involved intensive traditional tillage with multiple 3–5 passes (i.e., 1–2 tillage operations to incorporate the leftover crop stubble prior to planting, followed by 2–3 operations including tillage and leveling for puddling and seeding). In AT, *aman* rice in all cropping systems was established under puddled condition, while for winter and spring season crops, seeds were sown into ∼ 25 cm crop stubble, and crops were established under ST with residue retention. In CA, both monsoon and winter (*boro*) rice were established under non-puddled condition, while for other winter and spring crops, seeds were sown ∼ 25 cm crop stubble and established under ST. Details of the cropping systems and tillage treatments are provided in Table S1.

### 2.4 Crop and residue management

#### 2.4.1 Crop management

Variety, seed rate, spacing, and fertilizer rates for each crop in each cropping system are presented in Table S2. The transplanting date was ≈ 15 July ( ± 3 days) for monsoon rice and ≈ 15 January ( ± 3 days) for winter *boro* rice; the sowing date was ≈ 15 November ( ± 3 days) for maize and wheat and ≈ 15 February ( ± 3 days) for mung bean in double cropping (R-MB) system and March end to April in triple cropping (≈ 25 March in R-W-MB and ≈ 25 April in R-M-MB, respectively) (Fig. 2) systems. The monsoon rice varieties under double- and triple-cropping systems were BR-11 and BINA DHAN-7, respectively. Other varieties were BRRI DHAN-29 for *boro* rice, BARI GOM-26 for wheat, NK 40 for maize, and BARI Mung-6 for mung bean.

Fertilizers for each crop were applied according to rates recommended by the Bangladesh Agricultural Research Council (BARC), Fertilizer Recommendation Guide(FRG, 2018). Fertilizer rates for short-season transplanted *aman* season rice (*T. aman*), long-season *T.aman,* and *boro* rice were 68–22–25–30, 81–25–30–36, and 100–24–50–0 (N-P-K-S), respectively. Fertilizer rates for wheat and maize were 100–24–50–110 and 250–45–130–0 (N-P-K-S), respectively (Table S2). The same fertilizer rates, application, and management were followed across the systems and tillage practices in respective crops. For N, urea was applied at planting and 1–2 times as top-dressing. For P, K and S, diammonium phosphate, triple super phosphate, muriate of potash and gypsum were applied at planting, except for the second application of K to maize at tasseling. Weeds in the rice fields were controlled under CT and AT through puddling, tillage and manual weeding, but as part of CA, for rice and other crops, they were controlled by glyphosate and the pre-emergence herbicide Pretilachor. Due to the absence of pests and diseases, there was no need for pesticide application (see Table S1 for details).

*Aman* rice was grown as a rainfed crop, apart from about 180 mm of irrigation water applied for puddling in each season. In *boro* rice, 1186–1795 mm of irrigation water was applied depending on rainfall amount and distribution across the seasons, where CA and AT required 400 and 200 mm less water than CT, respectively. Under CA and AT, flood irrigation was applied to achieve soft soil two to three days prior to seedling transplanting. After rice seedling transplanting, frequent irrigation water was applied on a regular basis (3–4 days intervals) as in the conventional system to ensure proper crop establishment; then, irrigation was applied by alternate wetting and drying. In each plot, one perforated PVC pipe (30 cm long, up to 20 cm deep) was set up. The soil was then excavated from the pipe and daily water levels were measured. When the free-moving water level was more profound than 15 cm in any of the four replications, irrigation was applied to a flood depth of 7–8 cm above the soil surface, depending on the stage and height of the rice crop. In CT, however, full flood irrigation of at least 7–8 cm depth was applied regularly for up to 30 days after transplanting to maintain floodwater and then re-irrigated when hairline cracks were observed on the soil surface (Gathala et al., 2011). Irrigation was stopped two weeks before harvest.

In maize, depending on seasonal rainfall, up to five irrigations at five stages were applied with a total of 254–351 mm during the season: (1) at the three-leaf stage (V3), (2) at the six-leaf stage (V6) after fertilizer was banded, (3) at V10 after banding, (4) at silking, and (5) at the grain watery milking stage. In all tillage treatments in wheat, maximum of four irrigations were applied: (1) at crown root initiation (CRI), (2) at late tillering, (3) at flowering, and (3) at milking. On average, wheat crop received 115–189 mm across treatments. Mung bean relied on soil-stored water and rainfall apart from one pre-sowing light irrigation.

#### 2.4.2 Crop residue management

At each crop harvest, leftover crop residue quantity was measured in all treatment plots (Table S3). However, amount of crop residue retained and soil surface coverage as mulch depended on crops, cropping systems, and tillage options. Aman rice was harvested at the height of ≈ 25 cm in CA and AT and at ≈ 5 cm in CT. The succeeding winter crops were seeded/planted into about 3.0 t ha^−1^ crop residue as a surface mulch/stubble in CA and AT. In CT, about 0.70 t ha^−1^ crop residue was incorporated during land preparation. In R-W and R-R cropping systems, wheat and boro rice were harvested at ≈ 25 cm height which was about 2.5–3.5 t ha^−1^ crop residue retained as a mulch in CA plots, but in AT and CT, both crops were harvested at the height of ≈ 5 cm; this was incorporated before next aman rice. In CA, under the R-MB system, the whole mung bean residue (about 2.5 t ha^−1^) was retained in the field. The maize stover was harvested at ≈ 40 cm height in CA, which was more than 5 t ha^−1^, whereas, in AT and CT, maize was harvested at ≈ 5 cm height in the R-M system. In triple cropping systems (R-W-MB and R-M-MB), the winter crops (wheat and maize) were reaped at ≈ 25 and ≈ 40 cm, respectively, under CA and AT. In addition, whole stover of mungbean was retained in the field after final pod collection in CA and AT, but this was removed in CT. During the three years of experimentation, in CA under triple cropping systems (R-W-MB and R-M-MB), on average annual crop biomass 8.0 and 11.3 t ha^−1^, respectively, were recycled; this was varied from 5.4 to 9.0 t ha^−1^ in double cropping systems (Table S3).

### 2.5 Crop and systems grain, protein, and calorie productivity

#### 2.5.1 Yield sampling and measurements

The procedure for rice and wheat grain and straw yield sampling was similar. The samples for grain yield estimation were taken from the centre of the plots, from an area of 18 m^2^ in each plot. The harvested rice and wheat plants were bundled and left in the plots for thorough sun drying, after which the grain and straw samples were weighed, followed by threshing and weighing of the grain. Concurrently, grain moisture readings were taken using a moisture meter (Draminski Co., Olsztyn, Poland). Rice and wheat grain yields were adjusted to 14% moisture content. The pre-weighed straw sub-samples were dried in an oven for 72 h at 70 °C to a constant weight. The samples were then weighed with a precision balance.

Across all treatments, maize samples from each plot were taken from 5 m of 6 rows, with 1 m of row ends and each outside row excluded, giving a harvest area of 18 m^2^. All the cobs from the sampling area were harvested. Sampled cobs were thoroughly dried and weighed, after which they were shelled. A sub-sample of five representative cobs was collected for moisture determination. Maize yield was adjusted to 15.5% moisture content.

Mung bean samples for grain yield were taken from an 18 m^2^ area (6.0 m x 3.0 m). Due to the indeterminate growth habit of mung bean varieties, pods were harvested twice from each plot and straw yield recorded after the second picking. The pods were sun-dried, weighed, threshed, and grains cleaned. Grain moisture was measured using a moisture meter and yield was adjusted to 12% moisture content. In plots where neither pods nor grains had formed or matured, straw biomass was still recorded from a 2 m^2^ area. Straw biomass was weighed using a field balance and sub-samples were dried in an oven for 72 h at 70 °C, with dry weight recorded when constant weight was achieved.

#### 2.5.2 Computation of rice equivalent yield

The rice equivalent yield (REY) for the component crops of each cropping system was calculated from the price in Bangladesh currency ha^−1^ (BDT ha^−1^) as follows:

$$\mathrm{REY}\left(t{\mathrm{ha}}^{-1}\right)=\frac{\mathrm{grain}\mathrm{yield}\left(\mathrm{kg}{\mathrm{ha}}^{-1}\right)*\mathrm{price}\mathrm{of}\mathrm{respective}\mathrm{crop}/\mathrm{variety}\left(\mathrm{BDT}{\mathrm{kg}}^{-1}\right)}{\mathrm{price}\;\mathrm{of}\;\mathrm{BR}\;11\mathrm{rice}(\mathrm{BDT}{\mathrm{kg}}^{-1})*1000}$$

To calculate REY, the prices of respective individual crop/cultivar used in each cropping system in each year relative to the price of long-duration monsoon rice (cv. BR11). The respective crop of short-duration monsoon rice (cv. BINA DHAN-7), winter rice (BRRI Dhan-29), wheat, maize and mung bean prices, respectively and individual crop prices (Table 1) were used in their respective seasons. The equivalent rice yield for each system (i.e., SREY, t ha^−1^) was determined by summing the REY of individual crops for each cropping system or tillage treatment.

**Table 1:** Prices for various inputs and marketable outputs used during the three years of experiment in a sub-tropical environment, Jamalpur, Bangladesh.

**Input or output (prices in USD†)**		**2012–13**	**2013–14**	**2014–15**
**Input category**				
Labor	Wage (USD person-day^−1^)	3.10	3.10	3.10
Land use	USD ha^−1^ yr^−1^	274	274	274
Seed	Maize (USD kg^−1^)	5.95	5.95	5.95
	Wheat (USD kg^−1^)	0.42	0.42	0.42
	*Aman* rice (USD kg^−1^) short variety	0.24	0.24	0.24
	*Aman* rice (USD kg^−1^) long variety	0.24	0.24	0.24
	*Boro* rice (USD kg^−1^)	0.24	0.24	0.24
	Mung bean seed (USD kg^−1^)	0.89	0.89	0.89
Fertilizer	Urea (USD kg^−1^)	0.19	0.19	0.19
	TSP (USD kg^−1^)	0.26	0.26	0.26
	MoP (USD kg^−1^)	0.18	0.18	0.18
	Gypsum (USD kg^−1^)	0.18	0.18	0.18
	ZnSO_4_ (USD kg^−1^))	2.14	2.14	2.14
	Borax (USD kg^−1^)	0.00	0.00	0.00
Irrigation	Amount and application fee (USD hr^−1^)	0.89	0.89	0.89
Fuel	Diesel ( USD L^−1^)	0.81	0.81	0.81
Threshing and shelling	*Aman* rice (USD t^−1^ grain)	17.86	17.86	17.86
	*Boro* rice (USD t^−1^ grain)	17.86	17.86	17.86
	Wheat (USD t^−1^ grain)	23.81	23.81	23.81
	Maize (USD t^−1^ grain)	5.95	5.95	5.95
	Mung bean (USD t^−1^ grain)	11.90	11.90	11.90
Herbicide and pesticide	Glyphosate (USD litre^−1^)	10.48	10.48	10.48
	Afinity (USD litre^−1^)	17.86	17.86	17.86
	Pretilachor (USD litre^−1^)	10.71	10.71	10.71
	Tilt (USD litre^−1^)	14.88	14.88	14.88
	Furadon (USD kg^−1^)	1.49	1.49	1.49
**Crop**				
Maize	Grain price (USD t^−1^)	178.57	184.52	184.52
	Stover price (USD t^−1^)	8.93	8.93	8.93
	Cob price (USD t^−1^)	5.95	5.95	5.95
Wheat	Grain price (USD t^−1^)	238.10	238.10	238.10
	Straw price (USD t^−1^)	5.95	5.95	5.95
*Aman* rice (short duration)	Grain price (USD t^−1^)	196.43	196.43	196.43
	Straw price (USD t^−1^)	5.95	5.95	5.95
*Aman* rice (long duration)	Grain price (USD t^−1^)	178.57	178.57	178.57
	Straw price (USD t^−1^)	5.95	5.95	5.95
*Boro* rice	Grain price (USD t^−1^)	178.57	178.57	178.57
	Straw price (USD t^−1^)	8.93	8.93	8.93
Mung bean	Grain price (USD t^−1^)	714.29	714.29	714.29
	Stover price (USD t^−1^)	5.95	5.95	5.95

† conversion rate: 1 USD = 84 BDT.

#### 2.5.3 Computation of grain protein and grain calorie equivalent yields

In addition to REY, grain protein and calorie equivalent yields were also calculated using the grain protein and calorie conversion factors reported in Bangladesh’s Food Composition Tables (Shaheen et al., 2013). Grain protein of 6.6 g, 11.2 g, 9.9 g and 23.7 g per 100 g and grain calories of 345 Kcal, 344 Kcal, 344 Kcal and 351 Kcal per 100 g were used for rice, wheat, maize and mung bean, respectively, to calculate grain protein (kg ha^−1^) and grain calorie (Gcal ha^−1^) equivalent yields. Although protein and caloric values may change following grain processing and cooking, we present them as an indicator of potential protein and energy availability from each treatment. System protein and calorie equivalent yields were obtained from the sum of all component crops' protein or calorie yields for a cropping system. The relative changes in SREY, system protein and calorie yields, and systems gross margins were compared for CA and AT against CT and for different cropping systems against R-R system.

### 2.6 Economic analysis

All inputs required to produce a crop and economically valuable outputs were recorded during each cropping season. The total variable costs were considered for computing the total production cost. The variable costs included land use (land rent), labor use, tillage, machinery use (for planting), seed, fertilizer, agro-chemicals, irrigation, weeding, harvesting, and threshing. The human labor cost (based on 8-hour person-day) was accounted for land preparation, seeding/transplanting, irrigation, fertilizer and agro-chemicals application, intercultural operations (such as weeding and bundling), harvesting, and threshing and cleaning. The labor cost was computed using the labor wage rate of the research station where the field experiment was conducted. The irrigation cost was estimated as the total hours required for irrigation in each plot and then multiplied by the electricity unit (Kw h^−1^) and per unit electricity charge. Similarly, the time (h) required by a two- or four-wheel tractor-drawn machine to complete each field operation (such as tillage, seeding and threshing) was recorded and expressed as h ha^−1^; simultaneously the fuel consumption (L ha^−1^) in each plot for each operation was recorded. Gross returns (GR) were computed by multiplying the grain and straw/stover yield of each crop by the offered prices in the established local market, which varied from season to season (Table 1). Gross margins (GM) were calculated as the difference between GR and total variable cost (TVC). The systems GR, TVC and GM were calculated by adding together the associated costs and benefits of the harvested crops within a crop calendar year. All input and output prices are presented in Table 1.

The distributions of labor use and gross margin under different cropping systems were plotted with scatter quadrate charts. Based on labor use and gross margin, four cropping system groups were designated: (1) low labor use and low gross margin (low-low); (2) low labor use and high gross margin (low-high); (3) high labor use and high gross margin (high-high); and (4) high labor use and low gross margin (high-low).

### 2.7 Statistical analysis

Before performing statistical analysis, the normality assumption of analysis of variance (ANOVA) was checked by Shapiro and Wilk (1965) using the JMP statistical software (V11 software, Buckinghamshire, UK). The test for homogeneity of variance was also performed using the Bartlett's test (Snedecor and Cochran, 1989). There was no need for data transformation as the normality assumption of ANOVA was fully met. We followed the procedure to build statistical models for data analysis for split plot design in fixed plots, which is suggested for long-term experiments with the complexity of cropping system: CS (main-plot), tillage; T (sub-plot) and year (Y), together with within-year replication/block (R) (Onofri et al., 2016). We performed the serial correlation using a random-effects model, which accounted for the compound symmetry variance-covariance structure. The analysis progressed using a complete three-factor analysis of variance (ANOVA). The treatments – ‘CS’ and ‘T′ - were fixed effects and were randomly allocated to plots. ‘Year’ was a repeated factor; this was combined with the treatment model by introducing the term “Year + CSxY + TxY + CSxTxY”. ‘Replication’ was a randomized unit, so we kept it under random effect by including replication interactions with all treatments. The final model was tested using the JMP software.

Fixed effect: Y + CS + T + CSxY + TxY + CSxT + CSxTxY

Random effect: R + RxY + RxCS + RxCSxY + RxCSxT

All variable means were compared using Tukey’s honest signiﬁcant diﬀerence at p = 0.05, where significant treatment means were separated using alphabet letters.

## 3 Results

### 3.1 Crops and cropping systems grain, calorie, and protein yields

ANOVA indicated that the grain, calorie, and protein yields did not differ across years except for winter crops' calorie and protein yields (Table 2). Individual cropping system and tillage effects on grain, protein, and calorie yields were influenced significantly. Still, cropping system by tillage and cropping system by year interactions were significant for winter crop and cropping system yields, but not for monsoon rice. The year by tillage and year by cropping system by tillage interactions also did not affect yields significantly (Table 2).

**Table 2:** Effect of six cropping systems and three tillage options on rice equivalent grain yield, grain calorie, and grain protein for crops and cropping systems (2013–2016) in a sub-tropical environment, Jamalpur, Bangladesh.

* and * * significance level at 0.01 and 0.05; † values in parenthesis are actual grain yields of respective crops; § values in parenthesis {} are rice equivalent yields of spring mung bean crop; Means followed by a common letter within a column are not significantly different by the HSD-test (Tukey’s honestly significant difference) at the 5% level of significance; cropping systems: R-R = rice-rice, R-W = rice-wheat, R-M = rice-maize, R-MB = rice-mungbean, R-W-MB = rice-wheat-mung bean, R-M-MB = rice-maize-mung bean; tillage and crop establishment treatments: CA = conservation agriculture, AT = alternate tillage, CT = conventional tillage.

#### 3.1.1 Crops and cropping systems grain yields

In this study, the REY of monsoon rice, winter crops, and cropping systems remained unchanged by season. The monsoon REY of the short-duration rice variety (BINA DHAN −7) was lower by about 8% in the triple cropping systems (R-W-MB, R-M-MB) than the long-duration variety (BR11) in the double cropping systems (R-R, R-M, R-W, R-MB). Considering the winter season crop REY, the R-M system produced the highest maize REY while the R-MB system produced the lowest mung bean REY. Winter rice (*boro*) REY under the R-R system was 15% higher than the wheat REY, compared to the REYs under the R-W or R-W-MB system, but winter crops REYs in the R-R and R-W systems were significantly lower (by 41.7% and 63.5%, respectively) than the maize REY in the R-M and R-M-MB systems. Considering systems level REYs, all six cropping systems followed the order from highest to lowest: R-W-MB ≥ R-M ≥ R-M-MB > R-R ≥ R-W ≥ R-MB. The R-W-MB system REY was 5%, 11%, 30%, 43% and 45% greater than those of R-M, R-M-MB, R-R, R-W and R-MB systems, respectively. The R-R system consistently had a higher system REY than the R-MB system. Among the tillage treatments, CT and AT had significantly higher (by 8.7% and 4.3%, respectively) monsoon rice REYs than CA; however, under CA, compared to CT, the winter REY was 12% higher and system REY was 5% higher (Table 2).

**Fig. 3:**
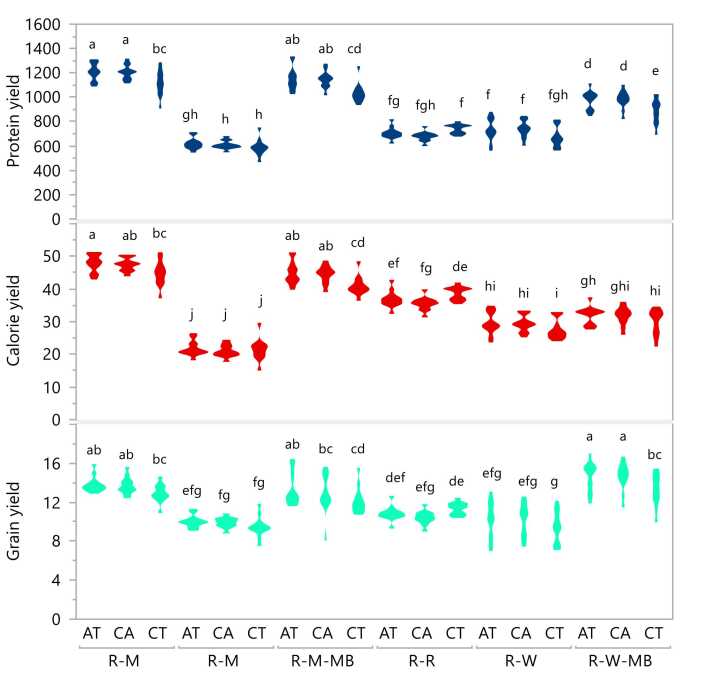
Interactive effects of cropping system and tillage options on system rice equivalent yields of grain (t ha^−1^), system calorie (Gcal ha^−1^), and system protein (kg ha^−1^) in a sub-tropical environment, Jamalpur, Bangladesh. CA, AT, and CT indicate conservation agriculture, alternate, and conventional tillage, respectively. R-R, R-W, and R-M stand for rice-rice, rice-wheat and rice-maize rotations, while R-MB, R-W-MB and R-M-MB indicate rice-mung bean, rice-wheat-mung bean, and rice-maize-mungbean rotations. The shape of the legend in respective treatment combinations shows the data distribution to across years and replications. The legends mean followed by a common letter horizontally are not significantly different by the HSD-test (Tukey’s honestly significant difference) at the 5% level of significance.

The cropping system by tillage interactions effect revealed that the REY of winter maize under CA and AT in R-M and R-M-MB rotations (8.8 t ha^−1^) was the highest, followed by CT under the same rotations (7.7 t ha^−1^). On the other hand, the REY of mung bean was lowest under CT in R-MB, followed by CT in wheat under R-W and R-W-MB rotations (Figure not shown). All cropping systems except R-R had the highest winter crop REY under CA, followed by AT. The yield increases of winter crops under CA compared to CT ranged from 12% to 18%, with the highest increase under R-W and the lowest under R-M and R-M-MB rotations. With AT, the yield increases of winter crops under different systems ranged from 11% to 14%. For R-R rotation, however, there was a 1% yield decrease under AT and a 4% decrease under CA. The R-M and R-W-MB rotations under CA and AT had the greatest systems-level yields (13.7–14.6 t ha^−1^), while the R-W and R-MB systems, regardless of tillage treatment, had the lowest (9.4–10.2 t ha^−1^) system yields (Fig. 3). In all cropping systems except R-R, CA and AT had higher systems REY (by 6–9%) than CT.

#### 3.1.2 Crops and cropping systems grain protein and calorie equivalent yields

Analysis of variance of the main effects of crop system and tillage showed a highly significant response to calorie and protein yields when both seasons and cropping systems were taken into consideration (Table 2). The grain calorie and grain protein yields of winter crops were higher by 4.5% and 4.9% in Year 1, respectively than in Year 2 or 3; this was due to better crop performance in Year 1.

Both grain calorie and grain protein yields followed almost similar trends as in REY for both seasons and cropping systems. In the monsoon rice season, the long-duration rice produced about 18.0% higher grain calorie and protein yields than shorter-duration rice. In winter crop season, maize had the highest calorie and protein yields and mung bean the lowest, with the former six times higher than the latter. Winter *boro* rice also produced 62% and 392% higher grain calories than wheat and mung bean grains, respectively. Similarly, while maize yielded the highest protein yield, mung bean yielded the lowest; wheat and winter rice achieved statistically similar protein yield. At the cropping systems level, grain calorie and protein yields respectively followed the order from highest to lowest: R-M > R-M-MB > R-R > R-W-MB = R-W > R-MB and R-M > R-M-MB > R-W-MB > R-W = R-R > R-MB. The R-M system consistently performed better in terms of both calorie and protein yields due to high yield potential of winter maize (Table 2), together with good protein content. The R-R system had the second highest calorie yield and R-W-MB the second highest protein yield, the latter due to the inclusion of the third crop in the system.

Tillage significantly influenced both grain calorie and protein yields of all crops in both seasons and for all cropping systems. CT yielded 8.4% and 7.8% greater calorie and protein yields in monsoon rice than in CA. However, this trend was quite opposite in winter crops and cropping systems, where CA provided higher calorie and protein yields, respectively, by 12.6% and 14.4% in winter crops and 3.0% and 6.4% in complete rotational systems (Table 2).

The interaction effects of the cropping system by tillage showed the highest grain calorie and protein yields (48 Gcal and 1208 kg ha^−1^, respectively) under CA and AT in the R-M system; the R-MB system had the lowest calorie and protein yields (21 Gcal and 606 kg ha^−1^, respectively) under all tillage treatments (Fig. 3). In both R-M and R-M-MB systems, the grain calorie and protein yields were significantly lower under CT than CA and AT. The increase in systems protein yield across cropping systems (except for R-R) ranged 3–10% under CA and 8–10% under AT compared to CT.

### 3.2 Crops and cropping systems economic analysis and labor use

#### 3.2.1 Production cost and gross margin

Production costs for both monsoon rice and different cropping systems were lowest in Year 3 compared to Years 1 and 2. (Table 3). In monsoon season, the production cost of the short-duration rice variety (BINA DHAN −7) was lower by about 7.5% in the triple cropping systems (R-W-MB, R-M-MB) than that of the long-duration variety (BR11) in the double cropping systems (R-R, R-M, R-W, R-MB) due to reduced labor and associated inputs required for short duration cultivars (Table 3). Of the winter crops, the winter *boro* rice had the highest production cost (USD 872 per ha^−1^); this was 123% higher than the lowest cost crop, mung bean (USD 391 per ha^−1^). The second highest production cost was for maize in the R-M and R-M-MB systems; these costs were about 19% lower than for winter rice but 59% and 86% higher than for wheat and mung bean. Across cropping systems, the R-M-MB system was the most input intensive, with its production cost higher than for R-R, R-W-MB, R-M, R-W and R-MB systems by about 3%, 7%, 14%, 41% and 50%, respectively. The introduction of mung bean in the R-W and R-M cropping systems incurred an additional 22% cost, equaling approximately USD 278 ha^−1^.

**Table 3:** Effect of six cropping systems and three tillage options on rice equivalent labor use, production cost, and gross margin for crops and cropping systems (2013–2016) in a sub-tropical environment, Jamalpur, Bangladesh.

Source	Cost of production (USD ha^−1^)			Gross margin (USD ha^−1^)			Labor use (Person days ha^−1^)		
	Monsoon crop	Winter crop	System	Monsoon crop	Winter crop	System	Monsoon crop	Winter crop	System
Year (Y)									
Year 1	650^a^	611^a^	1372^a^	276^a^	587^a^	881^a^	108^a^	81^a^	211^a^
Year 2	650^a^	599^a^	1358^a^	238^a^	571^a^	827^a^	107^ab^	76^a^	205^ab^
Year 3	596^b^	605^a^	1305^b^	289^a^	548^a^	862^a^	96^b^	77^a^	193^b^
Cropping system (CS)									
R-R	652^a^	872^a^	1524^b^	253^a^	240^c^	494^e^	107^a^	138^a^	245^a^
R-W	646^a^	460^c^	1106^e^	268^a^	399^b^	667^d^	105^ab^	39^d^	144^e^
R-M	646^a^	722^b^	1368^d^	288^a^	939^a^	1227^a^	105^ab^	73^c^	178^d^
R-MB	650^a^	391^d^	1041 ^f^	287^a^	449^b^	736^d^	106^a^	105^b^	211^c^
R-W-MB	600^b^	453^c^	1467^c^{414}§	260^a^	475^b^	1079^b^{344}§	100^bc^	36^d^	230^b^{94}§
R-M-MB	599^b^	733^b^	1563^a^	251^a^	909^a^	939^c^	99^c^	76^c^	209^c^
Tillage option (T)									
CA	617^c^	604^b^	1326^b^	245^b^	626a	902^a^	100^b^	75^c^	195^c^
AT	635^b^	614^a^	1356^a^	275^a^	595^b^	896^a^	104^a^	77^b^	202^b^
CT	645^a^	597^b^	1352^a^	284^a^	485^c^	773^b^	106^a^	81^a^	212^a^
Analysis of variance (*p*-values)									
Y	0.006 * *	0.292	0.002 * *	0.289	0.245	0.293	0.006 * *	0.089	0.003 * *
CS	< 0.001 * *	< 0.001 * *	< 0.001 * *	0.646	< 0.001 * *	< 0.001 * *	< 0.001 * *	< 0.001 * *	< 0.001 * *
T	< 0.001 * *	< 0.001 * *	< 0.001 * *	0.003 * *	< 0.001 * *	< 0.001 * *	< 0.001 * *	< 0.001 * *	< 0.001 * *
Y × CS	0.463	< 0.001 * *	< 0.001 * *	0.199	< 0.001 * *	0.002 * *	0.338	0.003 * *	< 0.001 * *
Y × T	0.005 * *	0.365	0.007 * *	0.406	0.172	0.285	0.002 * *	0.039 *	0.264
CS × T	0.023 *	< 0.001 * *	< 0.001 * *	0.934	< 0.001 * *	< 0.001 * *	0.012 *	< 0.001 * *	< 0.001 * *
Y × CS × T	0.596	< 0.001 * *	0.133	0.999	0.675	0.934	0.209	< 0.001 * *	0.010 *

* and * * significance level at 0.01 and 0.05; § values in parenthesis are rice equivalent of spring mung bean crop; Means followed by a common letter within a column are not significantly different by the HSD-test (Tukey’s honestly significant difference) at the 5% level of significance; R-R = rice-rice, R-W = rice-wheat, R-M = rice-maize, R-MB = rice-mungbean, R-W-MB = rice-wheat-mungbean, R-M-MB = rice-maize-mungbean; tillage and crop establishment treatments: CA = conservation agriculture, AT = alternate tillage, CT = conventional tillage.

**Fig. 4:**
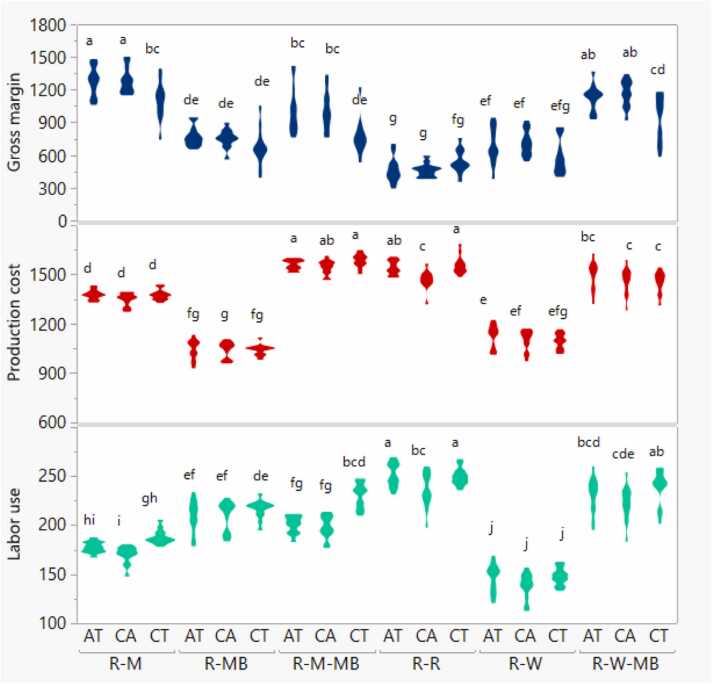
Interactive effects of cropping system and tillage option on winter labor use and gross margin, and system labor use (person days ha^−1^), production cost (USD ha^−1^) and system gross margin (USD ha^−1^) in a sub-tropical environment, Jamalpur, Bangladesh. CA, AT, and CT indicate conservation agriculture, alternate, and conventional tillage, respectively. R-R, R-W, and R-M indicate rice-rice, rice-wheat, and rice-maize rotations, while R-MB, R-W-MB, and R-M-BM indicate rice-mung bean, rice-wheat-mung bean, and rice-maize-mungbean rotations. The shape of the legend in the respective treatment combinations shows the data distribution across years and replications. The legends mean followed by a common letter horizontally are not significantly different by the HSD-test (Tukey’s honestly significant difference) at the 5% level of significance.

In all three tillage options, the production cost of monsoon rice was highest for CT and lowest for CA, whereas it was intermediate for AT. For winter crops, it was about USD 14 ha^−1^ higher in AT than in CT or CA, while at the cropping systems level, it was about 2% lower in CA than CT or AT (Table 3). The cropping system's interaction effect by tillage showed that the highest production cost was for R-R and R-M-MB systems grown under CT and lowest for R-MB with CA (Fig. 4). At systems level, the R-R system under CA had significantly lower production cost than under CT due to the elimination of puddling and less water requirement for crop establishment.

In general, gross margins of all crops and cropping systems were similar among years (Table 3). The gross margin for monsoon rice varied from USD 251 to USD 288 ha^−1^, but it did not differ among cropping systems. For winter crops, the highest gross margin was for maize in the R-M and R-M-MB rotations, followed by wheat and mung bean in the R-W, R-W-MB and R-MB rotations. The lowest gross margin was for winter rice in the R-R rotation; this was 66% lower than the gross margin for winter wheat in the R-W rotation. At the cropping systems level, the R-M rotation had the highest gross margin; this was higher than for the R-R, R-W, R-MB, R-M-MB and R-W-MB systems, by 149%, 84%, 67%, 31% and 14%, respectively. The difference in gross margins between the highest (R-M) and the lowest (R-R) system was USD 733 per ha^−1^.

The CT achieved a gross margin of about USD 39 ha^−1^ higher than CA in monsoon rice; however, CA had a higher gross margin than CT by USD 141 and USD 129 ha^−1^ from winter crops and different cropping systems, respectively. For both winter crops and different cropping systems, AT also had a higher gross margin than CT, by USD 100 and USD 113 ha^−1^, respectively. The interaction effects of cropping system by tillage demonstrated that the R-M system practised with CA resulted in the highest gross margin. In contrast, the R-R system practised with CA resulted in the lowest gross margin (Fig. 4). The gross margins were significantly higher in R-M, R-M-MB and R-W-MB systems by 16%, 32% and 22%, respectively, under CA and AT than CT, but tillage options did not influence in the rest of the cropping systems (R-MB, R-W and R-R). These results indicated that CA and AT practices are more profitable for the former than the latter systems.

#### 3.2.2 Labor use

The main differences in labor use between different cropping systems or tillage options are accounted for by differences in labor required for different crops under different tillage options in different seasons. The interaction effect of cropping system by tillage had a significant impact on labor use for both crop seasons and cropping systems (Table 3). Across the seasons, the number of person-days required for all crops and cropping systems was lower in Year 3 than in Year 1. For monsoon rice grown under different cropping systems, the short-duration variety (grown under the triple-cropped system) required six person-days ha^−1^ less than the long-duration variety (grown under the double-cropped system). Among the winter crops, the highest labor use (138 person-days ha^−1^) was required for winter rice cultivation, which was on average was higher by 33, 63 and 100 person-days ha^−1^ for mung bean, maize and wheat, respectively. At the cropping systems level, the R-R system had the highest labor use (245 person-days ha^−1^), followed by R-W-MB (230 person-days ha^−1^), and lowest for R-W (144 person-days ha^−1^). The intensification of the R-W to R-W-MB and R-M to R-M-MB systems required 41 additional person-days ha^−1^.

The number of labor person-days ha^−1^ under different tillage treatments differed with crop seasons and the cropping system. The total labor required for CA was fewer than for CT by 5, 6 and 17 person days ha^−1^ for monsoon rice, winter crops and cropping systems, respectively. AT also required fewer person-days ha^−1^ than CT for different winter crops and cropping systems. The interaction effect of the cropping system by tillage showed the highest number of person-days ha^−1^ required for the R-R system with crops grown under CT or AT, while the lowest number of person-days ha^−1^ was required for the R-W system with all tillage methods. In R-MB and R-W systems, the labor use didn’t make any difference among the three tillage options because of comparatively lower labor required for mung bean and wheat planting and land preparation in conventional system compared to other winter crops. Whereas winter maize and boro rice required more labor respectively for dibbling and seedling transplanting, CA-based practices reduced effective labor requirement for R-M, R-M-MB, R-R, and R-W-MB systems over CT (Fig. 4).

#### 3.2.3 Distribution of gross margin and labor use

**Fig. 5:**
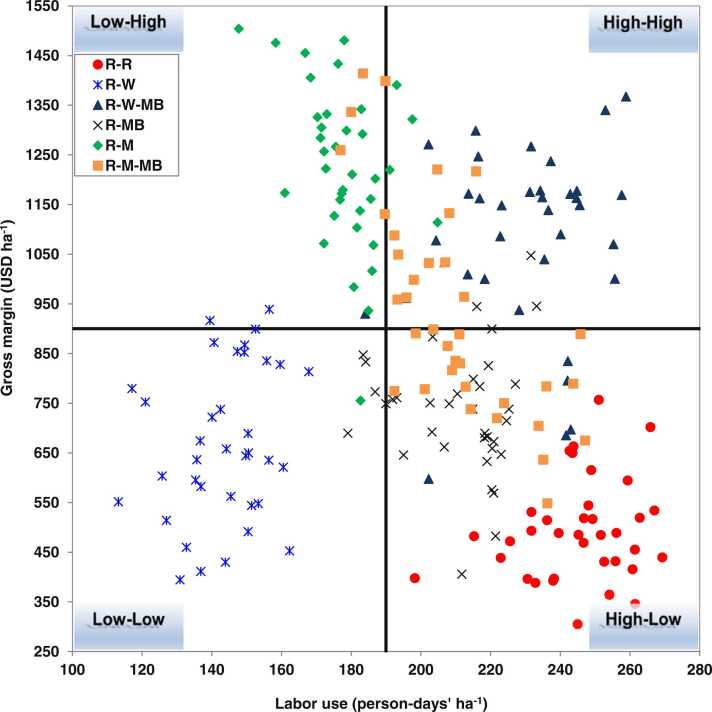
Distribution of gross margin in respect of labor used for different cropping systems. The distribution is divided into four categories according to each system’s gross margin and labor used with respect to cropping systems: Low-Low, Low-High, High-High, and High-Low represent low labor use and low-profit margin, low labor use and high-profit margin, high labor use and high-profit margin, and high labor use and low-profit margin, respectively. R-R, R-W, and R-M indicate rice-rice, rice-wheat, and rice-maize rotations, while R-MB, R-W-MB, and R-M-BM indicate rice-mung bean, rice-wheat-mung bean, and rice-maize-mung bean rotations.

The distribution of labor use and gross margin among six cropping systems is demonstrated in Fig. 5. The R-W rotation fell under the low-low group, with 100–190 person-days ha^−1^ labor use and 250–900 USD ha^−1^ gross margin. On the other hand, the R-M rotation fell under the low-high group, with labor use almost the same as for the low-low group but with a higher gross margin (900–1550 USD ha^−1^). The triple cropping systems R-W-MB and R-M-MB were grouped under high-high, indicating more labor intensive (190–280 person-days ha^−1^) but with high return (900–1550 USD ha^−1^); R-M-MB also overlapped with the high-low group. Finally, the R-R and R-MB rotations were grouped under the high-low group, indicating that these are more labor intensive (190–280 person-days ha^−1^) with lower gross margin (250–900 USD ha^−1^) (Fig. 5).

### 3.3 Relative change in CA and AT compared to CT and various rotations compared to R-R rotation

**Fig. 6:**
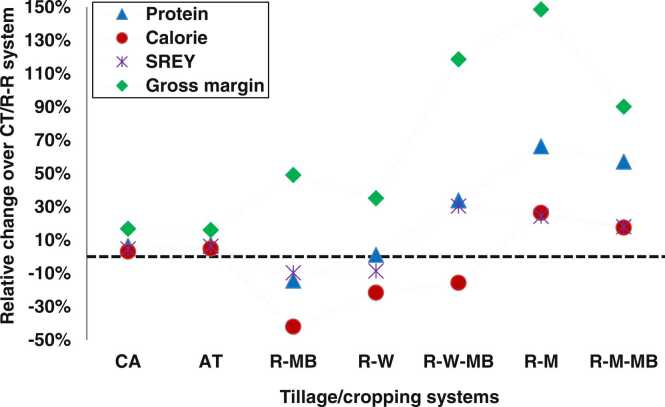
Relative change in system protein, system calorie, system rice equivalent yield (SREY), and system gross margin over conventional tillage/R-R system over three years of study. CA, AT, and CT indicate conservation agriculture, alternate, and conventional tillage, respectively. R-R, R-W, and R-M indicate rice-rice, rice-wheat, and rice-maize rotations, while R-MB, R-W-MB, and R-M-BM indicate rice-mung bean, rice-wheat-mung bean, and rice-maize-mung bean rotations.

The relative changes in SREY, system grain protein and calorie yields, and system gross margin over CT and R-R rotation compared to alternative tillage options (CA and AT) and cropping systems are presented in Fig. 6. The SREY increased under CA and AT by 4.6% and 6.1%, respectively, compared to CT. These higher yields, in combination with lower production costs under CA and AT, resulted in approximately 16% higher gross margin compared to CT. Regarding protein and calorie yields, the increase was about 7% and 4%, respectively, under CA and AT compared to CT. The change in SREY in the R-W-MB system compared to the R-R system was highest (30%) but negative (−8%) under the R-W system. There was a 24% increase in SREY under the R-M system compared to the R-R system. In contrast, the increase was lower under the R-M-MB system due to the inability of farmers to harvest mung bean seed because of the shorter growing window between maize and monsoon rice (Fig. 2). A negative grain yield change (−10%) and negative protein yield change (−14%) were also recorded for the R-MB system compared to the R-R system. The highest difference in protein yield (∼62%) was observed for the R-M and R-M-MB systems, followed by the R-W-MB system (34%) compared to the R-R system. In terms of calorie yield, only the R-M and R-M-MB cropping systems had a positive yield change, while the R-MB, R-W and R-W-MB systems showed negative changes compared to the R-R system. Likewise, compared to the R-R system, all the alternative cropping systems had positive changes in gross margins, with highest (148%) under R-M and lowest (35%) under R-W. When we further deepened the analysis on the combined effect of the tillage option by cropping system, SREY was higher by 55% and 42% in R-W-MB and R-M systems, respectively, with CA compared to R-W system with CT (Fig. 3). In terms of system protein and calorie yields, they were higher by 105% and 122% in the R-M system with CA compared to the R-MB system with CT. Likewise, the highest and lowest labor use was associated with CA in R-R and R-W systems, respectively, whereas the R-R system with CA used more than 112 person days ha^−1^ compared to the R-W system with CA. Similarly, the highest gross margin (178%) was achieved under the R-M system with CA while over the lowest was for R-R system with CA (Fig. 4).

## 4 Discussion

### 4.1 Effects on crops and cropping systems grain, calorie, and protein productivity

#### 4.1.1 Crops and cropping systems grain yield

In this study, monsoon REY was lower by about 8% in the triple cropping systems than in the double cropping systems due to the use of the short-duration BINA DHAN-7 rice variety in the former. This variety has lower yield potential because of fewer grains per panicle and lower crop biomass than the long-duration variety BR-11 (Tiongco and Hossain, 2015). In winter, both maize and *boro* rice produced higher crop biomass and grain yield compared to wheat (Table S3). Their higher yield potential could be attributed to significantly higher number of growing degree days due to longer crop seasons, fewer cloudy days, and higher amount of solar radiation (Timsina et al., 2010, Gathala et al., 2016). Considering systems REY, all cropping systems followed the order from highest to lowest: R-W-MB ≥ R-M ≥ R-M-MB > R-R ≥ R-W ≥ R-MB. Although the relatively low-yielding, short-duration rice cultivar was used in the R-W-MB rotation, the aggregate REY of this system was still higher due to the inclusion of mung bean in the rotation, which has a considerably higher market price (Table 1). The R-M system also produced higher system REY due to the use of the long-duration rice cultivar and the high potential productivity of the single cross maize hybrid (Timsina et al., 2010). The R-M-MB rotation did not produce a system yield higher than the R-M rotation despite the inclusion of short-duration mung bean because of its inability to set grains, as its grain-filling period coincided with the onset of the monsoon (Fig. 1). Consistent with our findings, Islam et al. (2019) also reported a higher yield from the R-W-MB rotation compared to the R-R, R-W and R-M rotations in several locations of the EGP. However, in contrast to our findings, Rashid et al. (2019) demonstrated that the R-M-MB rotation could be successfully grown under controlled conditions in clay loam soils in high rainfall areas in research station in northwest Bangladesh, though it would likely be difficult to disseminate to farmers’ field conditions. The R-R system consistently resulted in higher systems REY compared to the R-W or R-MB systems, but it requires intensive energy, an excessive amount of water and labor, and entails a high cost of production (Gathala et al., 2015, Gathala et al., 2016, Gathala et al., 2020a).

Traditionally, rice cultivation has been practiced under wet tillage (puddled) conditions, creating soft anaerobic soil. Most rice varieties are bred considering the suitability of seedling transplanting under wet tillage conditions. It is quite possible that such varieties do not perform equally well under non-puddled (or zero tillage) conditions (Chakraborty et al., 2017, Gathala et al., 2011 Jat et al., 2019b). Our results also confirmed that CT and AT (puddled for rice) had significantly higher (by 8.7% and 4.3%, respectively) monsoon REYs than CA among the tillage treatments. On the other hand, CA resulted in higher REYs (12%) for winter crops and different cropping systems than CT (Table 2). An increase in yield could be attributed to the continuous crop residue retentions on the soil surface as mulch (Supplementary table 3), which facilitates better growing conditions through more soil moisture conservation, buffering surface soil temperature, and advancing the planting. These results are consistent with recent synthesis studies in the EGP (Islam et al., 2019, Jat et al., 2020, Gathala et al., 2020b), where rice yields were reported to be either equal to, or lower under, CA than CT. However, these patterns were reversed for the succeeding dry season crops and rotational systems, with consistently higher yields under CA than CT.

Recent synthesis studies with various crop rotations in south Asia also demonstrate the potential of CA-based agronomic management for increasing yields of many crops and cropping systems except for rice; in these studies, the increment in grain yield varied depending on the layering of CA components over CT (Jat et al., 2020, Gathala et al., 2020b). Singh et al. (2016) reported higher R-M system yields in northwest India, and Islam et al. (2019) reported higher R-M and R-W system yields in eastern India under ZT compared to CT. In the R-R system, however, CT provided a yield higher by 5% compared to AT and 8% compared to CA. Higher systems yield under the R-R rotation is likely to be associated with soil organic matter build-up and nitrogen accumulation under the fully soil-saturated condition (Sharma and De Datta, 1986). Consistent with these findings, Islam et al. (2019) also reported higher systems yield under the R-R rotation compared to the R-W or R-M rotation under CT compared to CA. However, Yadav et al. (2017) reported higher grain and biomass yields in eastern India for the R-R system under no-tillage with 30% residue retention compared to CT with full residue incorporation. The rice yield reduction under CA was highly dependent on the soil type (texture), rainfall distribution, and land topography. The review of several studies established that heavier soils (clay to silty clay loam) had either lower or equal yield (−6.1 to 2.3%) under no-till (non-puddled) system compared to conventional puddled system than lighter soils (sandy loam to coarse sand) where rice yield reduction was about 10%. In the latter soils, the yield penalty was much higher due to higher percolation rate and low nitrogen use efficiency because of higher losses (Gathala et al., 2015, Chakraborty et al., 2017; Chaki et al., 2020). In our study, rice yield under CA had either equal or lower yield penalty (<5%), but this was compensated by higher yields of the succeeding dry season crops. This finding is consistent with Rashid et al. (2019) in northwest Bangladesh.

#### 4.1.2 Crops and cropping systems grain protein and calorie equivalent yields

In the recent decade, Bangladesh has consistently shown the sustainable food production growth and has achieved food self-sufficiency or food security in terms of per capita calorie availability (Farukh et al., 2020; National Food and Nutrition Security Policy, 2020). Despite this, the need for sustainable intensification and diversification of rice-based cropping systems under changing climate scenarios and ensuring the food and nutrition security of the increasing population will continue to remain the major challenge in Bangladesh agriculture (FAO, 2020). Our study can contribute to designing and developing the policy or strategies for achieving and sustaining food and nutritional security through sustainable crop intensification and diversification (Gathala et al., 2020a, Jat et al., 2020). In our study, the R-M system consistently performed better in calorie and protein yields due to high yield of winter maize (Table 2) and its high protein content (9.9%). The R-R system had the second highest calorie yield and R-W-MB the second highest protein yield. Higher calorie yield in the R-R system was due to the high-calorie content in rice grain (11.2%), while higher protein yield in the R-W-MB system was due to the high protein content in mung bean (23.1%) (Shaheen et al., 2013). Crop diversification, from R-R to R-M and R-W-MB systems, can provide balanced nutritional diets with higher protein and calorie (Jat et al., 2020, Jat et al., 2018).

Tillage significantly influenced the calorie and protein yields of all crops in both seasons and of all cropping systems. In monsoon rice, CT yielded 8.4% and 7.8% greater calorie and protein yields than CA. This trend was, however, quite the opposite for winter crops and cropping systems, where CA had higher calorie and protein yields, respectively, by 12.6% and 14.4% for winter crops and by 3% and 6.4% for cropping systems. These results agree with other findings in south Asia, where CA-based management practices have resulted in 3.0–6.0% higher protein yield for different cropping systems (Jat et al., 2018, Jat et al., 2020). The higher calorie and protein yields under CA and AT reflected higher systems grain yield in these management practices. Adopting suitable cropping systems in combination with the right technologies can improve the calorie and protein security of smallholder farmers in Bangladesh and south Asia (Islam et al., 2019, Gathala et al., 2020a, Jat et al., 2020). The R-M system practiced with CA provided the highest system-level grain, calorie, and protein yields. Maize is not the direct component of human diets in Bangladesh. However, this is a major source of poultry feed and chicken is one of the primary protein sources of human diets in the country. Therefore, there is a scope to include and promote maize in human diets by changing the consumers' dietary habits. Our findings confirm that adopting R-M and R-W-MB systems practiced with CA with optimum resource use can improve the food and nutritional security of the increasing population of Bangladesh. Since the R-M system requires higher amount of fertilizer compared to the R-R system (Supplementary Table 2), it might favor policy towards fertilizer subsidies and increase imports by the country. On the other hand, practicing the R-R system in the prime lands, where winter rice consumes about 1700 mm ha^−1^ water irrigated through groundwater sources, would also require high energy for extraction and thus would deplete the underground water.

### 4.2 Effect on crops and cropping systems profitability

In intensive cropping systems of the EGP, it is essential to know how smallholders can maximize their farm profits with the efficient and effective use of natural resources (land, water, energy, and labor). This study demonstrated the effects of six cropping systems and three tillage options on systems productivity and profitability. The R-M rotation resulted in a higher gross margin (approximately 150%) than the R-R rotation due to the high yields of long-duration hybrid maize grown in winter and long duration monsoon rice. Further, maize cultivation required lower labor and production costs (due to less irrigation water application) compared to boro rice. The higher gross margin of the R-M system compared to the R-R system is consistent with other studies in the EGP (Jat et al., 2014, Jat et al., 2020, Gathala et al., 2016, Gathala et al., 2021). A recent on-farm study spread over several hundred farmers in three countries of the EGP demonstrated that the R-M rotation would be the most profitable cropping system for smallholder farmers (Gathala et al., 2021). The triple cropped R-M-MB rotation resulted in a lower gross margin than the double cropped R-M rotation due to additional production cost required for mung bean cultivation. In addition, many farmers were unable to harvest mung bean timely and achieve economic yield due to its harvest time synchronizing with the monsoon rainfall. Although inclusion of mung bean in the R-M rotation lowered the profit due to the use of short-duration rice variety in rotation, such inclusion would be a good option from the soil sustainability point of view as it has potential to improve soil health (Gathala et al., 2015, Jat et al., 2018; Jat et al., 2019). The double cropped R-W and R-MB also resulted in approximately 42% higher gross margin compared to the R-R rotation (Fig. 4). This resulted mainly from lower labor use and production cost of mung bean compared to winter rice, and their higher market rice. When the R-W system was intensified with the inclusion of mung bean after wheat, it provided USD 247 ha^−1^ profit over the R-W system. These results are in conformity with Kumar et al. (2018), Gathala et al. (2020b), and Gathala et al. (2021). An additional 16% benefit was achieved when all five alternative cropping systems in the R-R system were layered with CA or AT management practices (Jat et al., 2020, Gathala et al., 2013, Gathala et al., 2020b, Kumar et al., 2018, Gathala et al., 2021).

The key criteria for selecting cropping systems in agriculture production systems are accessibility to labor and market, low labor wages, and higher gross margins (Emran et al., 2021). Labor accessibility in farming systems is becoming a major challenge due to outmigration of agricultural labor from the rural agrarian communities, seeking alternative livelihood opportunities (Sugden et al., 2014; Gathala et al., 2021) though due to the recent COVID-19 impact, there is some push backflow of labor in communities (Karim et al., 2020; Gatto et al., 2021; Talukder et al., 2021). Our study compared and grouped the six cropping systems into four groups through scattering quadratic graphics considering the labor and gross margins obtained (Fig. 5). The analysis showed R-M as the best cropping system since it required low labor hours but provided high income (low-high group) while R-R and R-MB were worse systems since they required high number of labor hours and provided low income (high-low group). Other four cropping systems fell under low-low (low labor use-low income) and high-high (high labor use-high income) groups indicating intermediate in preference. This kind of analysis will be helpful for policy and development leaders wanting to prioritize the different cropping systems in different agro-ecologies while considering accessibility to labor and wages. These results may also be helpful in the decision-making process of small holder farmers regarding where to invest their available resources efficiently, i.e., family labor to work in farm, or work off-farm to be able to pay the labor wages and irrigation water sources, etc.

The above distribution patterns may change in future with the adoption of agricultural machines and farm mechanization or labor dynamics. The Government of Bangladesh has recently been focusing on promoting agricultural mechanization throughout agricultural operations from seed to seed, such as land preparation and planting, intercultural operations, harvesting and threshing, and processing through a mega subsidy scheme ((National Agricultural Mechanization Policy, 2020) Mechanization in agriculture overcomes several problems and makes farms more profitable. Simultaneously, this also offers to solve secondary problems such as residue burning after combine harvest, over-tilling and puddling, over-exploitation of underground water, etc. (Shymsundar et al., 2019). As a result, in recent years, mechanized harvesting has been increasing in Bangladesh. The Government of Bangladesh should put a favorable policy environment for promoting CA-based sustainable intensification practices to mitigate the crop residue burning problem soon (Shymsundar et al., 2019; Jat et al., 2020; Gathala et al., 2020a). Immediate policy intervention is needed to ban crop residue burning. However, to manage the residues, an urgent need would be to access suitable machinery that could manage the crop residue in situ, like a Happy Seeder machinery developed and used in northwest India. This machine can plant/sow seed into crop residue of more than 10 t ha^−1^ without any problem in a single pass. Simultaneously, it also promotes straw management behind the combine to distribute evenly in the field (presently all combines accumulate crop residue in a small strip), which creates ideal conditions for operating any seeding machines (Yadvinder-Singh et al., 2020, Singh et al., 2021). It would also be good to encourage or incentivise farmers willing to manage crop residue in their fields, which will contribute to a clean environment, improve soil health, and conserve soil moisture by keeping mulch on the soil surface.

## 5 Conclusions

Increasing productivity and profitability and the long-term sustainability of cereal-based cropping systems remains a challenge for ensuring food and nutritional security of low to middle-income rural and urban populations in the EGP. This study showed that R-M and R-W-MB systems could increase systems REYs and systems grain calorie and protein yields by 50%, 5% and 27%, respectively, while providing 133% higher gross margins than the R-R system. An additional potential benefit could be harnessed when the five cropping systems (except the R-R system) studied here are grown using CA-based management practices. Our findings also indicated that the R-M system could result in a higher gross margin with lower labor use than the R-R system.

This study demonstrates that the application and adaptation of CA-based management practices can be beneficial for rice-based rotations practiced on loamy soil of a research farm located in a subtropical environment of the EGP in south Asia. Although the research farm is surrounded by farmers’ fields with similar climate and soil, crop management practices in research stations would differ from farmers’ fields with natural conditions and different socio-economic situations. Our findings offer a basket of technological interventions across the six cropping systems for smallholders to adopt as either a part of the component of CA or full package of CA (i.e., AT) depending on farmers’ priority and risk bearing capacity. Long-term studies are recommended to see the changes in insect pests and weed dynamics; soil biological, chemical, and physical properties; and adaptation and mitigation to climate change, etc., in farmers’ fields across soil types and climates. Particularly, it is important to evaluate and disseminate the promising cropping systems with CA or AT practices under farmers’ socio-economic conditions in Bangladesh and the EGP while considering farmers’ varying risk-bearing and investment capacities. The latter considerations are likely to be particularly important where efforts to extend these systems from research into real-world situations and adoption among smallholder farmers are the development goals in the EGP and south Asia as a whole.

## Declaration of Competing Interest

The authors declare that they have no known competing financial interests or personal relationships that could have appeared to influence the work reported in this paper.

## Data Availability

Data will be made available on request.
