# Supplementary material for: Reduced tillage and crop diversification can improve productivity and profitability of rice-based rotations of the Eastern Gangetic Plains
Source: Field Crops Res. 2023 Feb 1;291:108791. doi: 10.1016/j.fcr.2022.108791 (PMC9846111; doi:10.1016/j.fcr.2022.108791)
Supplement: Supplementary file 1 — Supplementary material. [file mmc1.docx]

Supplementary table 1. Crop establishment, residue and weed management details for six cropping systems with three tillage and crop establishment treatments in a subtropical environment, Jamalpur, Bangladesh.

|  | | **Conservation agriculture (CA)** | **Alternate tillage (AT)** | **Conventional tillage (CT)** |
| --- | --- | --- | --- | --- |
| **Rice-Rice (R-R) system** | | | | |
| Tillage, land preparation and planting | Aman | Unpuddled transplanting of rice seedlings into 25-30 cm of the retained anchored residue of the preceding winter crop (boro rice). The soil was saturated with rain or irrigation water and seedlings were transplanted on the following day. Due to the absence of puddling, transplanting occurred at least a week before the conventional tillage treatment. | Three full tillage passes in standing water with a power tiller into the leftover residue of preceding boro rice, followed by laddering. The soil was saturated with rain or irrigation water and rice seedlings were transplanted on the following day. | Three full tillage passes in standing water with a power tiller into the leftover residue of preceding boro rice, followed by laddering. The soil was saturated with rain or irrigation water and rice seedlings were transplanted on the following day. |
|  | Boro | Unpuddled transplanting of rice seedlings with 25-30 cm anchored residue retention of preceding aman rice. Before transplanting, soil was saturated by irrigation water for at least 48 hrs. Due to the absence of puddling, transplanting occurred at least a week before the conventional tillage treatment. | Same as for boro rice in CA. | After aman rice harvest, the one-two pass of dry tillage followed applied irrigation. Three full tillage passes in standing water with a power tiller into the leftover residue of preceding aman rice, followed by laddering. Soil was saturated with irrigation water and rice seedlings were transplanted on the following day |
| Residue management | Aman -Boro | After each crop*,* 25-30 cm of anchored rice residues were retained in the plots. | After aman rice*,* 25-30 cm of anchored rice residues were retained in the plots. Boro rice was harvested at 5 cm height from the ground and all straw (except 5 cm) was removed; the 5 cm of anchored residue was incorporated during aman rice puddling. | Each rice crop was harvested by cutting at 5 cm from the ground. All harvested straw was removed; the 5 cm height of anchored residue was incorporated during puddling in both crops. |
| Weed management | Aman | At least five days before planting, 1 kg active ingredient (a. i.) glyphosate ha^–1^ was applied by mixing in 320–400 L ha^–1^ of water and 0.15–0.2 kg urea ha^–1^ with a three-nozzle flat-fan spray boom. A pre-emergence herbicide Pretilachor @ 500 g a. i. in 400 L of water ha^-1^ was applied 1-3 days after transplanting. If any weeds were still observed, they were removed by uprooting. The soil was not turned over during manual weeding. | Weeds were generally controlled by puddling. If any weeds persisted, they were removed by uprooting. | Same as for aman rice in AT |
|  | Boro | Same as for aman rice in CA | Same as in CA | Same as for aman rice in CT. |
| **Rice-Wheat (R-W) system** | | | | |
| Tillage, land preparation and planting | Aman | Same as for aman rice in CA in R-R system | Same as for aman rice in AT in R-R system | Same as for aman rice in CT in R-R system |
|  | Wheat | Single pass seeding plus basal fertilizer application using strip tillage planter; retention of 25-30 cm height of anchored rice residues. As no prior tillage was needed, this treatment was seeded at least a week before the conventional tillage treatment. | Same as in CA | Two full tillage passes with a power tiller, followed by hand broadcasting of seed, and further by running a power tiller and then laddering. |
| Residue management | Aman- Wheat | After harvesting of each crop*,* 25-30 cm height of anchored residue of each crop was retained in the field. | After aman rice*,* 25-30 cm of anchored rice residues were retained in the field. After wheat, only 5 cm of anchored wheat residues were incorporated during aman rice puddling and all other straw was removed from the field. | After harvest*,* only 5 cm of anchored residues of each crop were incorporated in the plots while all other straw was removed from the field. |
| Weed management | Aman | Same as for aman rice in CA in R-R system | Same as for aman rice in AT in R-R system | Same as for aman rice in CT in R-R system |
|  | Wheat | At least five days before planting, 1 kg a. i. glyphosate ha^–1^ together with 0.15–0.2 kg urea ha^–1^ was mixed in the sprayer tank with 320-400 L water and applied to increase the efficiency of plant absorption of herbicide. Within two days after seeding, weeds were further controlled through a pre-emergent application of Pendimethalin @ 1 kg a.i. ha^–1^ in 320–400 L of water. Herbicides are more effective if soil is slightly moist. Therefore, when rainfall occurred, herbicides were applied immediately after the rain. If the broad-leaved weeds appeared, they were controlled between 25 and 30 days after application of post-emergence with Carfentrazone-Ethyl (Affinity) @ 20 grams a. i. ha^–1^. Each herbicide was applied with a three-nozzle flat-fan spray boom. If any weeds persisted, they were removed by uprooting. | Same as in CA | Weeds were controlled by mechanical tillage and by hand weeding, wherever necessary. Weeding was done prior to fertilizer application. |
| **Rice-Maize (R-M) system** | | | | |
| Tillage, land preparation and planting | Aman | Same as for aman rice in CA in R-R system | Same as for aman rice in AT in R-R system | Same as for aman rice in CT in R-R system |
|  | Maize | Same as for wheat in CA in R-W system | Same as in CA | Same as for wheat in CT in R-W system |
| Residue management | Aman-Maize | After aman rice*,* 25-30 cm of anchored rice residue was retained in the maize plots. After maize, 40 cm of anchored maize residues were retained in the aman rice plots. | After aman rice*,* 25-30 cm of anchored rice residues were retained in the maize plots. After maize harvest, the stover was cut at 5 cm height and the anchored stover was retained in rice plots. All stover (except 5 cm height) was removed from the field. | After harvest*,* residues of only 5 cm in height of each crop were incorporated and all other straw was removed from the field. |
| Weed management | Aman | Same as for aman rice in CA in R-R system | Same as for aman rice in AT in R-R system | Same as for aman rice in CT in R-R system |
|  | Maize | Same as for wheat in CA in R-W system. | Same as in CA | Same as for wheat in CT in R-W system |
| **Rice-Mungbean (R-MB) system** | | | | |
| Tillage, land preparation and planting | Aman | Same as for aman rice in CA in R-R system | Same for aman rice in AT in R-R system | Same for aman rice in CT in R-R system |
|  | Mungbean | Same as for wheat in CA in R-W system | Same as in CA | Same as for wheat in CT in R-W system |
| Residue management | Aman-Mungbean | After aman rice*,* 25-30 cm of anchored rice residues were retained in the mungbean plots. After mungbean, all anchored mungbean residues were retained in the aman rice plots. | After aman rice*,* 25-30 cm of anchored rice residues were retained in the mungbean plots. After mungbean harvest, all stover was removed from the field. | After harvest*,* residues of each crop from only 5 cm in height were retained while all other stover was removed from the field. |
| Weed management | Aman | Same as for aman rice in CA in R-R system | Same as for aman rice in AT in R-R system | Same as for aman rice in CT in R-R system |
|  | Mungbean | Same as for maize in CA in R-M system | Same as in CA | Weeds were controlled by 1-2 manual weeding. |
| **Rice-Wheat-Mungbean (R-W-MB) system** | | | | |
| Tillage, land preparation and planting | Aman | Same as for aman rice in CA in R-R system | Same for aman rice in AT in R-R system | Same as for aman rice in CT in R-R system |
|  | Wheat | Same as in wheat in CA in R-W system | Same as for wheat in AT in R-W system | Same as for wheat in CT in R-W system |
|  | Mungbean | Same as in CA in R-MB system | Same as in AT in R-MB system | Same as in CT in R-MB system |
| Residue management | Aman-Wheat-Mungbean | After aman rice*,* 25-30 cm height of anchored rice residues were retained in the wheat plots. After wheat, 30 cm height of anchored wheat residues were retained in the mungban plots. After mungbean, all anchored mungbean residues were retained in the rice plots. | After rice harvest*,* only 25-30 cm height of anchored rice residues were retained in the wheat plots; for rice and mungbean, residues of only 5 cm height of previous crops were retained. | After harvest*,* residues of only 5 cm in height of each crop were retained in the subsequent crops while all other stover was removed from the field. |
| Weed management | Aman | Same as for aman rice in CA in R-W system | Same as for aman rice in AT in R-W system | Same as for aman rice in CT in R-W system |
|  | Wheat | Same as for wheat in CA in R-W system | Same as for wheat in AT in R-W system | Same as for wheat in CT in R-W system |
|  | Mungbean | Same as for mungbean in CA in R-MB system | Same as for mungbean in AT in R-MB system | Same as for mungbean in CT in R-MB system |
| **Rice-Maize-Mungbean (R-M-MB) system** | | | | |
| Tillage, land preparation and planting | Aman | Same as for aman rice in CA in R-R system | Same for aman rice in AT in R-R system | Same as for aman rice in CT in R-R system |
|  | Maize | Same as for maize in CA in R-M system | Same as for maize in AT in R-M system | Same as for maize in CT in R-M system |
|  | Mungbean | Same as for mungbean in CA in R-MB system | Same as mungbean in AT in R-MB system | Same as for mungbean in CT in R-MB system |
| Residue management | Aman-Maize-Mungbean | After aman rice*,* anchored rice residues of 25-30 cm height were retained in the maize plots. After maize, 40 cm anchored maize residues were retained in the mungban plots. After mungbean, all anchored mungbean residues were retained in the rice plots. | After rice harvest*,* anchored rice residues of 25-30 cm height were retained in the maize plots; for rice and mungbean, residues of only 5 cm height of previous crop were retained. | After harvest*,* residues of only 5 cm height of each crop were retained in the subsequent crops while all other stover was removed from the field. |
| Weed management | Aman | Same as for aman rice in CA in R-M system | Same as for aman rice in AT in R-M system | Same as for aman rice in CT in R-M system |
|  | Maize | Same as for maize in CA in R-M system | Same as for maize in AT in R-M system | Same as for maize in CT in R-M system |
|  | Mungbean | Same as for mungbean in CA in R-MB system | Same as for mungbean in AT in R-MB system | Same as for mungbean in CT in R-MB system |

Supplementary table 2. Crop and fertilizer management for component crops under six cropping systems in a subtropical environment, Jamalpur, Bangladesh.

| Practices | Cropping system |  | Rice-rice | Rice-maize | Rice-wheat | Rice-mungbean | Rice-wheat-mungbean | Rice-maize-mungbean |
| --- | --- | --- | --- | --- | --- | --- | --- | --- |
| Variety | Crop 1* |  | BR-11 | BR-11 | BR-11 | BR-11 | BINA-7 | BINA-7 |
|  | Crop 2 |  | BRRI DHAN-29 | NK40 | BARI GOM-26 | BARI Mung-6 | BARI GOM-26 | NK40 |
|  | Crop 3 |  | _ | _ | _ | _ | BARI Mung-6 | BARI Mung-6 |
| Seed rate (kg ha^-1^), parenthesis showed the number of plants ha^-1^ | Crop 1 |  | 40 | 40 | 40 | 40 | 40 | 40 |
|  | Crop 2 |  | 40 | 20 (83333) | 120 | 25 | 120 | 20 (83333) |
|  | Crop 3 |  | _ | _ | _ | _ | 25 | 25 |
| Spacing (cm) | Crop 1 |  | 20 x 20 | 20 x 20 | 20 x 20 | 20 x 20 | 20 x 20 | 20 x 20 |
|  | Crop 2 |  | 20 x 20 | 60 x 20 | 20 | 30 | 20 | 60 |
|  | Crop 3 |  | _ | _ | _ | _ | 30 | 30 |
| Transplanting/sowing date | Crop 1 |  | Jul-15 | Jul-15 | Jul-15 | Jul-15 | Jul-15 | Jul-15 |
|  | Crop 2 |  | Jan-15 | Nov-15 | Nov-15 | Feb-15 | Nov-07 | Nov-07 |
|  | Crop 3 |  | _ | _ | _ | _ | March-25 | April-25 |
| N fertilizer rate (kg ha^-1^) | Crop 1 | Amount | 81 | 81 | 81 | 81 | 68 | 68 |
|  |  | Splits | one-third each at 7-8 DAT, 27-28 DAT, and at PI | Same as R-R | Same as R-R | Same as R-R | Same as R-R | Same as R-R |
|  | Crop 2 | Amount | 100 | 250 | 100 | 0 | 100 | 250 |
|  |  | Splits | one-third each as basal, 25-30 DAT and 45-50 DAT | 80 kg as basal, 90 kg at V6, 80 kg at V10 | Two-third as basal, one-third at 20 DAS | 0 | Two-third as basal, one-third at 20 DAS | 80 kg as basal, 90 kg at V6, 80 kg at V10 |
| P fertilizer rate (kg ha^-1^) | Crop 1 | Amount | 25 | 25 | 25 | 25 | 22 | 22 |
|  |  | Splits | All as basal | All as basal | All as basal | All as basal | All as basal | All as basal |
|  | Crop 2 | Amount | 24 | 45 | 24 | 0 | 24 | 45 |
|  |  | Splits | All as basal | All as basal | All as basal | 0 | All as basal | All as basal |
| K fertilizer rate (kg ha^-1^) | Crop 1 | Amount | 30 | 30 | 30 | 30 | 25 | 25 |
|  |  | Splits | 20 kg basal; 10 kg at PI | 20 kg basal; 10 kg at PI | 20 kg basal; 10 kg at PI | 20 kg basal; 10 kg at PI | 16 kg basal; 9 kg at PI | 16 kg basal; 9 kg at PI |
|  | Crop 2 | Amount | 50 | 130 | 50 | 0 | 50 | 130 |
|  |  | Splits | All as basal | 80 kg as basal, 50 kg at V10 | All as basal | 0 | All as basal | 80 kg as basal, 50 kg at V10 |
| S fertilizer rate (kg ha^-1^) | Crop 1 | Amount | 36 | 36 | 36 | 36 | 30 | 30 |
|  |  | Splits | All as basal | All as basal | All as basal | All as basal | All as basal | All as basal |
|  | Crop 2 | Amount | 0 | 0 | 110 | 0 | 110 | 0 |
|  |  | Splits | 0 | 0 | All as basal | 0 | All as basal | 0 |

*Crops represented as Crop1= monsoon season (July to November; rice); Crop2 = winter dry season (November to May; rice, wheat and maize); Crop3 = Spring season (April to June; mung bean)

Supplementary table 3. The amount of crop residue recycled under different cropping systems and tillage options as a surface mulch or incorporation during three years in different crop seasons.

| Cropping systems | Tillage options | 2013-14 | | | 2014-15 | | | 2015-16 | | | Total crop residue recycled in three years |
| --- | --- | --- | --- | --- | --- | --- | --- | --- | --- | --- | --- |
|  |  | Aman rice residue into winter crops | Winter crops residue into Aman rice/mung bean | Mung bean residue into Aman rice | Aman rice residue into winter crops | Winter crops residue into Aman rice/mung bean | Mung bean residue into Aman rice | Aman rice residue into winter crops | Winter crops residue into Aman rice/mung bean | Mung bean residue into Aman rice |  |
|  | t ha^-1^ | | | | | | | | | | |
| RR | CA | 2.94 | 3.49 | - | 2.93 | 3.52 | - | 3.24 | 3.38 | - | 19.51 |
|  | AT | 3.26 | 0.72 | - | 2.91 | 0.78 | - | 3.05 | 0.70 | - | 11.41 |
|  | CT | 0.63 | 0.75 | - | 0.59 | 0.80 | - | 0.67 | 0.69 | - | 4.13 |
| RW | CA | 3.30 | 2.58 | - | 3.04 | 2.60 | - | 2.68 | 2.13 | - | 16.34 |
|  | AT | 3.60 | 0.50 | - | 2.93 | 0.52 | - | 2.83 | 0.40 | - | 10.78 |
|  | CT | 0.70 | 0.45 | - | 0.61 | 0.43 | - | 0.55 | 0.36 | - | 3.11 |
| RM | CA | 3.04 | 4.19 | - | 3.15 | 7.30 | - | 2.74 | 6.61 | - | 27.03 |
|  | AT | 3.66 | 0.72 | - | 3.03 | 1.25 | - | 2.77 | 1.11 | - | 12.54 |
|  | CT | 0.69 | 0.75 | - | 0.61 | 1.13 | - | 0.61 | 1.06 | - | 4.84 |
| RMB | CA | 3.21 | 2.44 | - | 3.43 | 2.39 | - | 2.85 | 2.83 | - | 17.15 |
|  | AT | 3.30 | 0.24 | - | 3.22 | 0.23 | - | 3.20 | 0.27 | - | 10.47 |
|  | CT | 0.70 | 0.20 | - | 0.67 | 0.20 | - | 0.58 | 0.26 | - | 2.61 |
| RWMB | CA | 2.55 | 2.79 | 2.28 | 2.40 | 2.88 | 2.23 | 2.53 | 2.51 | 2.61 | 22.78 |
|  | AT | 2.55 | 2.65 | 2.37 | 2.67 | 2.79 | 2.33 | 2.65 | 2.52 | 2.50 | 23.03 |
|  | CT | 0.55 | 0.56 | 2.13 | 0.50 | 0.42 | 2.09 | 0.54 | 0.45 | 2.18 | 9.42 |
| RMMB | CA | 2.41 | 6.53 | 1.98 | 1.92 | 7.45 | 0.92 | 2.39 | 7.65 | 2.63 | 33.89 |
|  | AT | 2.63 | 6.21 | 1.90 | 2.16 | 7.19 | 1.05 | 2.47 | 7.32 | 2.58 | 33.52 |
|  | CT | 0.53 | 0.88 | 1.52 | 0.43 | 1.03 | 0.82 | 0.54 | 1.09 | 2.06 | 8.91 |

Supplementary table 4. Effect of season and tillage and crop establishment practices on rice equivalent yield (REY) and yield components in a sub-tropical environment, Jamalpur, Bangladesh.

| **Interventions** | **Effective tillers (m^-2^)** | **Filled grains (panicle^-1^)** | **Unfilled grains (panicle^-1^)** | **1000-grain weight (g)** | **Biomass (t ha^-1^)** | **Grain yield (t ha^-1^)** |
| --- | --- | --- | --- | --- | --- | --- |
| **Monsoon rice (Aman)** | | | | | | |
| Year | | | | | | |
| Year 1 | 229.2^b^ | 110.5^a^ | 28.7c | 26.5^a^ | 11.0^a^ | 4.8^a^ |
| Year 2 | 220.8^b^ | 89.0^b^ | 42.0^b^ | 24.1^b^ | 10.3^b^ | 4.7^ab^ |
| Year 3 | 244.4^a^ | 80.7^b^ | 33.6^b^ | 23.3^b^ | 10.3^b^ | 4.6^b^ |
| Tillage | | | | | | |
| CA | 230.7^a^ | 91.5^a^ | 36.3^a^ | 24.6^a^ | 10.1^b^ | 4.5^b^ |
| AT | 232.3^a^ | 92.3^a^ | 34.4^a^ | 24.6^a^ | 10.6^a^ | 4.8^a^ |
| CT | 231.4^a^ | 96.4^a^ | 33.6^a^ | 24.7^a^ | 10.8^a^ | 4.9^a^ |
| **Winter rice (Boro rice)** | | | | | | |
| Year | | | | | | |
| Year 1 | 373.4^a^ | 97.0^c^ | 16.2^b^ | 20.6^b^ | 13.0^a^ | 5.7^b^ |
| Year 2 | 266.0^b^ | 123.5^b^ | 23.3^a^ | 22.5^a^ | 13.2^a^ | 5.6^b^ |
| Year 3 | 249.3^b^ | 136.9^a^ | 21.7^a^ | 21.7^a^ | 13.1^a^ | 6.2^a^ |
| Tillage | | | | | | |
| CA | 296.4^a^ | 118.9^a^ | 20.6^a^ | 21.5^a^ | 12.6^a^ | 5.7^a^ |
| AT | 294.9^a^ | 118.5^a^ | 20.9^a^ | 21.3^a^ | 13.2^a^ | 5.9^a^ |
| CT | 297.4^a^ | 119.9^a^ | 19.7^a^ | 22.0^a^ | 13.5^a^ | 6.0^a^ |
| **Wheat** |  | **Grains (spike^-1^)** |  |  |  |  |
| Year | | | | | | |
| Year 1 | 330.81^a^ | 48.19^a^ |  | 44.69^a^ | 9.17^a^ | 3.98^a^ |
| Ye^a^r 2 | 321.63^a^ | 47.25^a^ |  | 45.91^a^ | 8.93^a^ | 3.89^a^ |
| Year 3 | 309.36^a^ | 47.07^a^ |  | 38.52^b^ | 8.80^a^ | 3.63^b^ |
| Tillage | | | | | | |
| CA | 340.24^a^ | 48.57^a^ |  | 43.18^a^ | 9.54^a^ | 4.10^a^ |
| AT | 331.41^a^ | 48.43^a^ |  | 43.52^a^ | 9.19^a^ | 3.92^a^ |
| CT | 290.16^b^ | 45.52^b^ |  | 42.41^a^ | 8.18^b^ | 3.48^b^ |
| **Maize** | **Cob length (cm)** | **Grains cob^-1^** |  |  |  |  |
| Year | | | | | | |
| Year 1 | 14.81^a^ | 312.71^a^ |  | 419.91^b^ | 20.61^a^ | 8.66^a^ |
| Year 2 | 15.23^a^ | 324.32^a^ |  | 439.69^a^ | 19.88^a^ | 8.09^b^ |
| Year 3 | 14.21^b^ | 313.68^a^ |  | 440.57^a^ | 20.09^a^ | 8.66^a^ |
| Tillage | | | | | | |
| CA | 15.44^a^ | 335.44^a^ |  | 434.44^a^ | 21.34^a^ | 8.96^a^ |
| AT | 15.06^a^ | 325.99^a^ |  | 434.62^a^ | 20.95^a^ | 8.82^a^ |
| CT | 13.77^b^ | 289.28^b^ |  | 431.11^a^ | 18.28^b^ | 7.64^b^ |
| **Mung bean** | **Plant density (m^-2^)** | **Grains pod^-1^** |  |  |  |  |
| Year | | | | | | |
| Year 1 | 45.08^a^ | 9.18^a^ |  | 45.89^a^ | 3.37^a^ | 1.10^a^ |
| Year 2 | 43.73^ab^ | 9.10^a^ |  | 45.43^a^ | 3.31^a^ | 1.08^a^ |
| Year 3 | 41.04^b^ | 9.32^a^ |  | 44.77^a^ | 3.68^a^ | 1.11^a^ |
| Tillage | | | | | | |
| CA | 46.85^a^ | 9.02^a^ |  | 45.66^a^ | 3.63^a^ | 1.17^a^ |
| AT | 44.77^a^ | 9.42^a^ |  | 44.89^a^ | 3.58^a^ | 1.14^a^ |
| CT | 38.23^b^ | 9.16^a^ |  | 45.54^a^ | 3.16^b^ | 0.98^b^ |

Means followed by a common letter within a column are not significantly different by the HSD-test (Tukey’s honestly significant difference) at the 5% level of significance;; tillage and crop establishment: CA = conservation agriculture, AT = alternate tillage, CT = conventional tillage.
